# Task Context Influences Brain Activation during Music Listening

**DOI:** 10.3389/fnhum.2017.00342

**Published:** 2017-06-29

**Authors:** Andjela Markovic, Jürg Kühnis, Lutz Jäncke

**Affiliations:** ^1^Division Neuropsychology, Institute of Psychology, University of ZurichZurich, Switzerland; ^2^International Normal Aging and Plasticity Imaging Center, University of ZurichZurich, Switzerland; ^3^University Research Priority Program, Dynamic of Healthy Aging, University of ZurichZurich, Switzerland

**Keywords:** EEG oscillation, music, heart rate, electrodermal response, music listening, EEG, music rating

## Abstract

In this paper, we examined brain activation in subjects during two music listening conditions: listening while simultaneously rating the musical piece being played [Listening and Rating (LR)] and listening to the musical pieces unconstrained [Listening (L)]. Using these two conditions, we tested whether the sequence in which the two conditions were fulfilled influenced the brain activation observable during the L condition (LR → L or L → LR). We recorded high-density EEG during the playing of four well-known positively experienced soundtracks in two subject groups. One group started with the L condition and continued with the LR condition (L → LR); the second group performed this experiment in reversed order (LR → L). We computed from the recorded EEG the power for different frequency bands (theta, lower alpha, upper alpha, lower beta, and upper beta). Statistical analysis revealed that the power in all examined frequency bands increased during the L condition but only when the subjects had not had previous experience with the LR condition (i.e., L → LR). For the subjects who began with the LR condition, there were no power increases during the L condition. Thus, the previous experience with the LR condition prevented subjects from developing the particular mental state associated with the typical power increase in all frequency bands. The subjects without previous experience of the LR condition listened to the musical pieces in an unconstrained and undisturbed manner and showed a general power increase in all frequency bands. We interpret the fact that unconstrained music listening was associated with increased power in all examined frequency bands as a neural indicator of a mental state that can best be described as a mind-wandering state during which the subjects are “drawn into” the music.

## Introduction

The capacity to appreciate music is a universal human phenomenon that helps inspire individual and social life. Neuroscientific studies have shown that music is processed in a cascade of steps that begins with segregation within the auditory stream, followed by the extraction and integration of a variety of acoustic features, and leading to cognitive memory-related processes that induce personal, often emotional, experiences. In the past 25 years, a wealth of brain-imaging studies has explored critical components of music and how they are processed in the underlying neural networks [e.g., chords (Tervaniemi et al., 2011), musical syntax (Koelsch, 2009), major/minor keys (Virtala and Tervaniemi, 2017), consonance/dissonance (Virtala and Tervaniemi, 2017), timing (Istók et al., 2013), absolute pitch (Hirata et al., 1999; Schaal et al., 2015), harmony (Passynkova et al., 2005), rhythm (Grahn and Rowe, 2009), and timbre (Meyer et al., 2006; Alluri et al., 2012) (for a summary see Jäncke, 2008; Koelsch, 2012)].

A major question in neuroscientific music research is whether musical pieces evoke specific subjective emotional and arousal reactions, which are related to neurophysiological activation patterns. A simple and often-used strategy in this context is to ask subjects to rate the musical pieces. Rating can be done either using questionnaires, which are applied after the presentation of the musical pieces, or by having subjects continuously rate the musical piece as it is played. These ratings can be made either verbally or along several dimensions (e.g., relaxation vs. arousal and/or sadness vs. happiness). The continuous rating of musical pieces is a relatively new strategy that has aided the identification of the time course of subjective experiences during music listening (Hutcherson et al., 2005; Mikutta et al., 2012, 2014; Jancke et al., 2015; Trost et al., 2015). For example, Mikutta et al. (2014) instructed subjects to move a computer mouse forward when they experienced increased arousal caused by the music, independently of their affective valence. The subjects were instructed to move the mouse backward when they experienced decreased arousal. The ratings were recorded and stored for offline analysis with a 100-Hz sampling rate. A slightly different strategy was used by Jancke et al. (2015). In their experiment, subjects rated the musical piece according to valence and arousal using analog scales (a vertical line for valence and a horizontal line for arousal). The subjects were instructed to click on the positions of the valence and arousal lines that best represented their actual valence and arousal ratings, respectively. As with Mikutta et al. (2014), these ratings were recorded for offline analysis with a sampling rate of 100 Hz. These continuous ratings have been applied in three different ways so far: (1) continuous rating of the musical piece simultaneously during neurophysiological recording [Listening and Rating (LR)] (Hutcherson et al., 2005); (2) continuous rating during a second presentation of the musical piece, without neurophysiological recording (LR without neurophysiological recording in a second separate session) (Mikutta et al., 2012, 2013, 2014; Jancke et al., 2015; Trost et al., 2015); (3) continuous rating during the first presentation of the musical piece, without neurophysiological recording (LR without neurophysiological recording in an initial separate session).

Although these approaches have yielded some insight into subjective emotional reactions and the related neurophysiological underpinnings, they are nevertheless associated with methodological problems. When continuously rating a musical piece while listening to it, the cognitive and motor processes are active, and are inactive during passive music listening (Hutcherson et al., 2005). Subjects must continuously self-monitor their feelings and thus must direct their attention not only to the musical piece but also to their own feelings. Basically, this is a multitasking situation during which the subject must: (1) listen attentively to the musical piece, (2) monitor their feelings, (3) transform subjective feelings into psychological categories, and (4) indicate these categories with motor responses. Thus, several psychological functions must be supervised and orchestrated. During passive and attentive listening, the subject has the opportunity to listen to a musical piece unconstrained by any other task. This provides the opportunity to be “drawn” into the music without external distraction. Thus, incoming stimuli other than the musical piece are inhibited and the music has the power to elicit a particular brain activation pattern that most likely can induce the extraordinary feelings and subjective experiences encountered when listening to music. This music experience is definitively different from one in which the listener is required to continuously rate the musical piece to which they are listening.

In addition, directing attention to one’s own emotions might also influence the subjective emotions and the associated neurophysiological and vegetative reactions. Some researchers suggest that focus on one’s own emotions may activate processes that might alter or modulate the emotional response (on both the neural and subjective level). As outlined by Hutcherson et al. (2005), three competing hypotheses can be derived regarding the effect of focusing on emotion on subjective, neural, and vegetative responses, which could be: (1) amplified, (2) weakened, or (3) uninfluenced. The findings based on these hypotheses have largely been inconsistent, supporting any one of the hypotheses (for a summary, see Hutcherson et al., 2005). However, the last fMRI paper on this topic revealed that subjective rating of ongoing emotional responses during music listening did not “decrease either self-reported experience of emotion or neural activations relative to passive viewing in any brain regions” (Hutcherson et al., 2005). However, relative to passive viewing, the act of rating increased activity in brain areas involved in the control of executive functions and emotions (e.g., anterior cingulate, insula).

Neural responses to musical pieces have been studied in the last 50 years using methods ranging from EEG/MEG to fMRI and PET. Although these methods have supported our understanding of the neural underpinnings of music listening, they are associated with their respective advantages and disadvantages. fMRI measurements during music listening are particularly problematic for several methodological and psychological reasons: (1) the obtrusive and unavoidable scanner noise induces undesirable activations in the auditory system (Herrmann et al., 2000; Novitski et al., 2006); (2) subjects have to suppress their processing of the background scanner noise in order to focus on the music stimuli, thus additional executive functions that are inactive during undisturbed music listening have to be activated; (3) the scanner environment is uncomfortable and frequently associated with negative emotions such as discomfort, claustrophobia, pain, low-level anxiety, and other variants of negative emotion (Heinrich et al., 2014; Keulers et al., 2014; Mutschler et al., 2014); (4) the fixation of the subject’s head in the coil is often associated with feelings of malaise and pain; (5) the background scanner noise is often so obtrusive that it greatly disturbs the aesthetic enjoyment of music. The latter especially has largely been neglected in fMRI-based music research, given that we do much to exploit modern methods (e.g., sophisticated HiFi presentation methods) and/or specific presentation environments (opera hall with world-class orchestra) to derive maximum enjoyment from music listening. Music presentations in fMRI environments are definitively of much lower quality than the music presentations to which we normally listen. Other studies have used PET to study blood flow responses to musical stimuli (Satoh et al., 2006). Although PET measurements are silent, they are associated with tracer injections into the subject’s blood, which is a stressful intervention for many subjects, thus most likely mitigating the enjoyment of music listening. Thus, fMRI and PET environments are definitively not the perfect experimental environments in which to study neural and emotional responses to aesthetically appealing music stimuli.

EEG and MEG, on the other hand, measure neurophysiological responses in an ecologically valid setting (e.g., sitting on a chair while listening to HiFi music without interfering noise). The relative disadvantage of EEG recordings is the lower spatial resolution (if the aim is to estimate the underlying cortical sources of EEG activity), although the correspondence between EEG-based estimations of intracortical sources and fMRI measurements is astonishingly high (Britz et al., 2010; Van de Ville et al., 2010). Several published studies have used EEG to measure neural responses during music listening (Petsche et al., 1993; Iwaki et al., 1997; Sarnthein et al., 1997; Bhattacharya and Petsche, 2001; Bhattacharya et al., 2001a,b; Altenmuller et al., 2002; Jaušovec and Habe, 2005; Baumgartner et al., 2006; Jausovec et al., 2006; Peterson and Thaut, 2007; Sammler et al., 2007; Schaefer et al., 2009, 2011a,b, 2013; Mikutta et al., 2012, 2014; Wu et al., 2012; Jancke et al., 2015; Jancke and Alahmadi, 2016; Rogenmoser et al., 2016). Some of these studies focused on the functional network characteristics during music listening and identified specific network features in various frequency bands. A further set of studies focused on the frontal activation asymmetry patterns during the listening of differently valenced and arousing music (Tsang et al., 2001; Altenmuller et al., 2002; Mikutta et al., 2012, 2014).

In the context of this paper, the study by Schaefer et al. (2011b) is of particular interest. The authors reported increased alpha band power during imagery of listening to a musical piece as compared to during the perception of the musical piece. The authors explained this increase during imagery as an indicator of a modulation of the attentional network. They related their finding to Klimesch’s “inhibition concept,” where alpha band oscillations are a neurophysiological indicator of an active inhibition of non-task-relevant cortical areas. Thus, when directing attention internally (as when imagining listening to the musical piece), numerous networks, especially those processing incoming information, must be inhibited. However, several studies have shown that alpha band power is increased also while listening to rhythms, tone sequences, and even natural musical pieces. In a previous study, we identified increased power during music listening not only in the alpha band, but also in the theta and beta bands (Jancke et al., 2015). We interpreted this general synchronizing in different frequency bands as a neural indicator of a psychological process during which the subject is torn into the music.

However, the question arises as to whether this general synchronization only occurs during unconstrained music listening or whether it is disrupted or diminished when music listening is accompanied by a task that must be performed simultaneously. In this context, we reconsidered the question of whether simultaneous music listening and rating (LR condition) will induce different neural and vegetative activations compared to passive listening without rating (listening: L condition). We were also interested in examining whether the sequence in which these experimental conditions are followed is important. Particularly, we were interested in whether the LR condition influences the subsequent L condition in terms of the neural, vegetative, and subjective responses. Thus, it is possible that when the subjects have rated the musical pieces first (LR condition), they might implicitly do so even when not required (L condition) subsequently. To measure the neural responses, we focused (similarly as in our previous paper) on the power in the theta, alpha, and beta bands. In addition, we used subjective ratings of the musical pieces and heart rate (HR) and electrodermal (EDA) responses. The HR and EDA were used because these measures are good indicators of vegetative arousal responses during music listening (Jancke et al., 2015; Koelsch and Jancke, 2015). Using these measures, we addressed the following research questions:

(1) Does the same synchronization occur in the theta, alpha, and beta bands during the L condition as we have shown previously (Jancke et al., 2015)?
(2) Does the LR condition diminish the synchronization in the different frequency bands?
(3) Is there less synchronization in the different frequency bands during the L condition when subjects have previously followed the LR condition? In other words, does the earlier experience of explicitly rating a musical piece prevent or negatively influence the synchronization of EEG oscillations?
(4) Are the HR and EDA responses different during the LR and L conditions?
(5) Are the HR and EDA responses during the L condition different when subjects have followed the LR condition prior?

## Materials and Methods

### Subjects

Fifty-one subjects (18 men and 33 women) took part in this experiment. All were students enrolled in psychology, biology, medicine, or computer sciences at the University Zurich or ETH Zurich. Mean age was 24 years (range: 19–31 years). All subjects were consistently right-handed, as revealed by the Edinburgh Handedness Inventory (Oldfield, 1971). In order to control for general cognitive abilities, we applied two short intelligence tests, namely the KAI (Kurztest der aktuellen geistigen Leistungsfähigkeit; Lehrl et al., 1992) and the MWT (Mehrfachwahl-Wortschatz-Intelligenz; Lehrl et al., 1974). These tests revealed above average general cognitive abilities for the participating subjects (KAI-IQ: mean = 124.02, *SD* = 12.08; MWT-IQ: mean = 109.57, *SD* = 13.17). To control for personality traits and emotional responsiveness subjects had to work on a personality questionnaire (NEO-FFI; Borkenau and Ostendorf, 1993), an alexithymia scale (TAS-26; Kupfer et al., 2001), and a rating scale measuring emotional responsiveness [Skalen zum Erleben von Emotionen (SEE); Becker, 2004]. The musical aptitudes of the participants were estimated using the Advanced Measure of Music Audiation (AMMA) test published by Gordon (1989). This procedure is based on the assumption that a fundamental prerequisite for musical aptitude is the ability to hold musical sounds in memory and detect melodic and rhythmic variations. During the AMMA test, the volunteers listened to short pairs of piano tone sequences and had to decide whether these sequences were equivalent, rhythmically different, or tonally different. The subjects scored above average (per the norms of the Gordon test; total score for all subjects: 56.16, *SD* = 20.31; mean and SD of the norm population for non-musicians = 50.6 ± 7.9). No subjects reported a history of present or past neurological, psychiatric, or audiological disorders, and all possessed an unremarkable audiological status. In addition, the subjects completed the Barcelona Music Reward Questionnaire (BMRQ), which is known to be a reliable measure of inter-individual variability in music-induced reward (Mas-Herrero et al., 2013). All subjects denied consuming illegal drugs or regular medication. None of the subjects indicated any history of professional musical training, as assessed by an in-house questionnaire frequently used by our research group. In addition, all subjects indicated that they did not perform in an orchestra, band, or choir within the last 5 years. Each subject received 50 Swiss Francs for the participation. The study was carried out in accordance with the Declaration of Helsinki principles, approved by the ethics committee of the University of Zurich. All subjects gave written, informed consent and were informed of their right to discontinue participation at any time.

The subjects were randomly assigned to one of the two groups. 26 subjects were assigned to group 1 (G1) while the remaining 25 subjects were allocated to group 2 (G2). There was no between groups difference with respect to the above-mentioned control variables (age, KAI, MWT, NEO-FFI, TAS-26, AMMA, SEE, and handedness).

### Stimuli

We used four musical pieces taken from well-known Oscar awarded sound tracks: (1) “Ship at Sea” from the “Pocahontas” sound track composed by Alan Menken; (2) “Pretty Peppy” from the soundtrack “The Artist” composed by Ludovic Bource; (3) “Concerning the Hobbits” from the soundtrack “Lord of the Rings” composed by Howard Shore; (4) “A Familiar Taste” from the soundtrack “The Social Network” composed by Trent Reznor and Atticus Ross. We have chosen these musical pieces according to a procedure applied by Lin et al. (2010). In a pilot study 20 students (not taking part in the final experiment) rated 20 musical pieces according to valence and arousal. Based on their evaluations we chose the above-mentioned musical pieces because of their dynamic variability in terms of arousal and valence ratings. We used the iTunes version of these musical pieces and transformed the mp4 format into the widely used mp3 format. The musical pieces were presented via HiFi earphones (Sennheiser, CX-350, Colchester, Essex, United Kingdom) with a convenient volume level (intensity = 75 dB). The duration of each musical piece was set to 3 min. Presentation of the musical pieces as well as the supervision of the entire experiment was controlled by Presentation software (Neurobehavioral Systems, Inc., Berkeley, CA, United States).

### Procedure

Over the entire course of the study, participants were seated in a comfortable chair in a sound-shielded room in front of a computer monitor. After fixation of the Geodesics EEG net, all subjects conducted an eyes open (EO) and eyes closed (EC) EEG measurement condition for 3 min each. During these conditions the subjects were instructed to relax and to let their mind wander. After these initial measurements, they practiced the usage of the computer mouse for the later following ratings for 6 min (3 min without and 3 min with music). After that the experimental conditions started.

We used two experimental conditions during which the four musical pieces (3 min duration each) were presented in random order. Between each musical piece a pause of 10 s duration was placed during which the subjects were instructed to relax. In one condition the subjects were instructed to listen passively to the musical pieces as they normally do when they listen to musical pieces for recreational purposes [Listening (L)]. During the second condition, the subjects were asked to continuously rate the musical pieces using the computer mouse. Thus, they simultaneously performed the rating task while listening to the musical pieces [Listening and Rating (LR)]. These ratings were done on a computer monitor placed in front of the subjects, on which they were shown a horizontal line for valence and a vertical line for arousal. The SAM for valence ranged from a frowning, unhappy figure (left) to a smiling, happy figure (right). The subjects were instructed to place/move the mouse cursor to the position of this horizontal valence line that best represents their actual valence rating. The values for each position on the valence line ranged from -1000 (left = unhappy) to +1000 (right = happy). Thus, values > 0 point to positive valence while values < 0 point to negative valence. For arousal rating we used a vertically arranged analog scale ranging from 0 (bottom = relaxed) to 1000 (top = excited). These rating data were collected with a sampling rate of 500 Hz. Twenty-six subjects started with the passive listening condition (G1) while the second group (G2) started with the LR condition. During the music presentation, we also collected EDA and HR. EDA and HR were recorded with a Biopack MP100 amplifier. The sampling rate for the vegetative data was 200 Hz. The subjects were asked not to clench their teeth and to avoid any kinds of movements other than mouse movement during the entire recording time. The entire experiment lasted about 1.5 h including fixing of electrodes, instruction, conducting the EEG measurements during the two listening conditions, debriefing and removing of electrodes.

We would like to explicitly mention that we refrained from employing further experimental conditions for this study. For example, it would have been possible to use a L-L and a LR-LR condition. However, we refrained from using them because in our previous study (Jancke et al., 2015) we showed that during repeated presentation of a musical piece the EEG and vegetative responses are strikingly similar when the subjects employ similar listening attitudes. Thus, we assumed that these conditions would unnecessarily expand the experiments and would have placed too much burden on the subjects.

### EEG Recording and Data Reduction

Electroencephalograms were recorded using a high-density Geodesics EEG system (GSN300; Electrical Geodesics, Inc., Eugene, OR, United States) with a 128-Channel HydroCel Geodesic Sensor Nets@ (HCGSN120). Data was sampled at 500 Hz and bandpass filtered at 0.1–30 Hz. The vertex electrode (Cz) served as an on-line reference. Impedance was maintained below 50 kOhm. For the exact positioning of the onset of music in the EEG, a marker channel was used to indicate the start and end of the musical piece. EEG analysis was conducted to identify the spectral correlates of music-induced fluctuations in cortical activations.

EEG data were analyzed with the *Brain Vision Analyzer* version 2.0.1 (Brain Products GmbH, D-82205 Gilching). In a first step, raw EEG data were bandpass filtered (1–30 Hz) including a notch-filter of 50 Hz to eliminate even very small oscillations leaking above 30 Hz. Eye movements and muscle artifacts were corrected by applying independent component analysis (Delorme et al., 2007). In addition, remaining muscle artifacts were identified and eliminated using ASR (The Artifact Subspace Reconstruction Method developed and programmed by Christian A. Kothe), a new algorithm designed to remove non-stationary high-variance signals from EEG time series and reconstruct the missing data using a spatial mixing matrix (assuming volume conduction). The EEG data of all channels were recomputed to average reference and frequency transformed by means of a fast Fourier transform (FFT).

### Analysis of Vegetative Data

From the time course of vegetative data, we calculated the mean HR and EDA for a one second lasting period before music presentation. These means were used as baseline measures. The HR and EDA values measured during music presentation were related to these baseline values resulting in percent change measures. The mean percent change measures for HR and EDA were subjected as dependent variables to subsequent statistical analysis. Here we focus on the mean HR and EDA response since these vegetative measures are known to be valid indicators of vegetative responses in the context of emotional reactions (Koelsch and Jancke, 2015).

### Main Data Analysis

For the artifact-free EEG data we computed spectral amplitudes (μV^2^/Hz) for the entire EEG recorded during each musical piece, and the EO and EC condition for the theta band (4–8 Hz), the lower alpha band (alpha-1: 8–10 Hz), the upper alpha band (alpha-2: 10–12 Hz), a lower beta band (beta-1: 13–20 Hz), and an upper beta band (beta-2: 20–30 Hz). In this paper we focus on the average EEG response during the entire music presentation in order to uncover general and tonic brain activations induced by the musical pieces in the different conditions. In order to gain statistical power, we defined nine electrode clusters of interest (EOI) for which the mean power of the frequency bands was calculated: three frontal, three central, and three parietal (left, midline, and right). The EOIs comprised the following sensors in the Geodesics space (the number indicate the particular Geodesics scalp electrodes):

(1) left frontal (LF) = 23 (F3), 24, 26, 27, and 33 (F7);
(2) midline frontal (MF) = 4, 5, 10, 11 (Fz), 12, 16, 18, and 19;
(3) right frontal (RF) = 2, 3, 122 (F8), 123, and 124 (F4);
(4) left central (LC) = 36 (C3) and 41;
(5) midline central (MC) = 31, Cz, and 80;
(6) right central (RC) = 103 and 104 (C4);
(7) left parietal (LP) = 47, 51, 52 (P3), 58, and 59;
(8) midline parietal (MP) = 61, 62 (Pz), and 78;
(9) right parietal (RP) = 91, 92 (P4), 96, 97, and 98.

These EOIs were chosen because they symmetrically cover the frontal, central, and parietal scalp regions of both hemispheres. Applying Geodesics EEG montages, several papers have used similar or even the same electrodes of interest (e.g., Curran and Dien, 2003). The power values of the different frequency bands from these EOIs were log transformed to stabilize the variances. These values were collapsed over the four different songs since they are all rated as being positive and arousing and the scope of this analysis was not to identify differences between the songs. We conducted four-way ANOVAs with three repeated measurements factors [**Region**: Frontal, Central, Parietal; **Hemisphere**: Left, Central, Right; **Condition**: Listening (L), Listening and Rating (LR)] and one grouping factor (Group: Group1: Listening → Listening and Rating, Group2: Listening and Rating → Listening). Arousal and valence ratings were subjected to *t*-tests comparing both groups (Group1: L → LR, Group2: LR → L). The HR and EDA measures were subjected to three-way ANOVAs with two repeated measurements factor (**Condition**: Listening, Rating and Listening; **Song**: Song 1 to Song 4) and one grouping factor (**Group**: Group1: L → LR, Group2: LR → L). For calculating the ANOVAs we used the *afex* R package for mixed and repeated measurements designs (Singmann et al., 2016). We only report Greenhouse–Geisser corrected *p*-values. A *p*-value of < = 0.05 was defined as significant. In case of significant interactions, we calculated Bonferroni–Holm-corrected *post hoc t*-tests as provided by the *lsmean* and *multcomp* R packages.

## Results

### Subjective Ratings and Vegetative Measures

The *t*-tests for the valence and arousal ratings revealed no between-group differences (all *p*-values > 0.3). The three-way ANOVA for HR revealed a significant main effect for **Condition** [*F*(1,52) = 5.87, *p* = 0.02, η^2^ = 0.02], which is qualified by HR decreases during the LR condition, while there was a slight HR increase during the L condition. There was no further significant main effect nor interaction with respect to the HR changes. The ANOVA for the EDA measures only revealed a trend toward a significant interaction between **Group and Condition** [*F*(1,52) = 3.03, *p* = 0.09, η^2^ = 0.006]. This interaction is qualified by differently changing EDA measures for both groups in the LR and L conditions. Those subjects starting with the L condition demonstrated reduced EDA values during the LR compared to the L condition. Subjects starting with the LR condition demonstrated slightly increased EDA measures during the LR condition.

### EEG Measures

**FIGURE 1:**
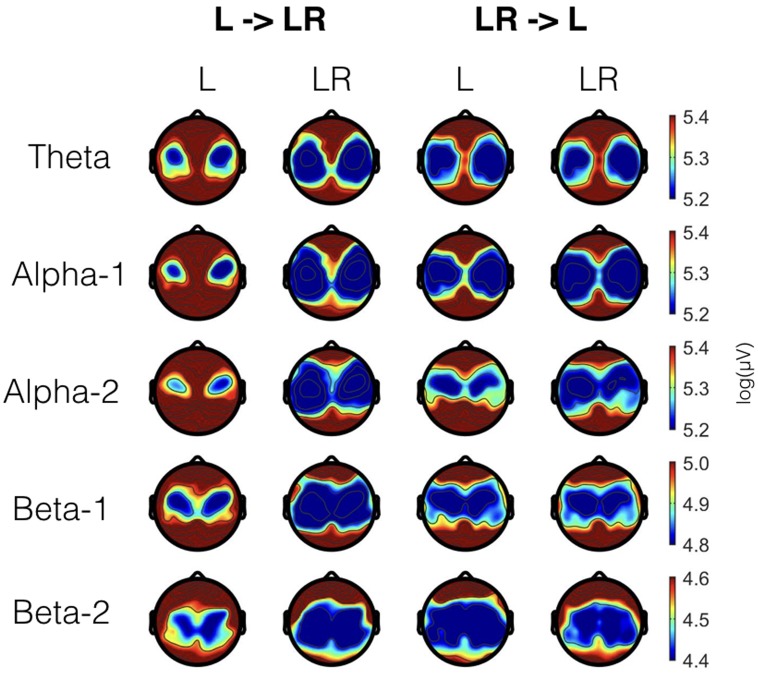
EEG topoplots for the different frequency bands broken down for the two conditions (L and LR) and the two groups (L → LR and LR → L). For each frequency band, we have used a slightly different scale. These scales are the same as for the **Figures 2**–**4**.

In **Figure 1** EEG topoplots are shown for each frequency band broken down for the major conditions (L and LR) and for the two groups with the different sequences of L and R (L → LR and LR → L). **Table 1** lists all significant effects obtained from the mixed ANOVAs for the five frequency bands. The detailed tabulation of these results is given in the Appendix at the end of this manuscript including the degrees of freedom and the general effect size measures. Here, we will only discuss the findings based on the reported *p*-values.

**Table 1:** Significant effects from the mixed ANOVAs for the different frequency bands.

Effect	Theta	Alpha1	Alpha2	Beta1	Beta2
Group	–	–		–	–
Condition	0.05	0.0004	<0.0001	<0.0001	–
Group:Condition	0.002	0.02	0.02	<0.0001	<0.0001
Hemisphere	<0.0001	<0.0001	0.05	–	0.01
Group:Hemisphere	–	–	–	–	–
Region	<0.0001	<0.0001	<0.0001	<0.0001	<0.0001
Group:Region	–	–	–	–	–
Condition:Hemisphere	–	–	0.03	–	–
Group:Condition:Hemisphere	0.05	–	–	–	–
Condition:Region	0.0007	<0.0001	<0.0001	<0.0001	–
Group:Condition:Region	0.06	0.09	–	–	–
Hemisphere:Region	<0.0001	<0.0001	<0.0001	0.009	–
Group:Hemisphere.Region	–	–	–	–	–
Condition:Hemisphere:Region	0.03	0.08	0.002	0.002	–
Group:Condition:Hemisphere:Region	–	–	–	–	–


**Indicated are the *p*-values based on Greenhouse–Geisser corrected estimations obtained from the *afex* R package.**

**FIGURE 2:**
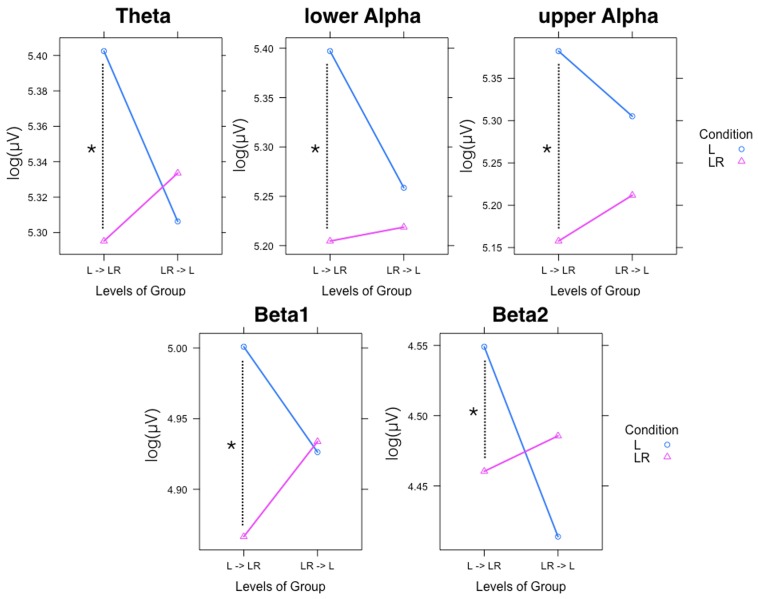
Significant two-way Group × Condition interactions for all frequency bands. Abscissa depicting the levels for the two groups: L = Group starting with Listening and continuing with Listening and Rating; LR = Group starting with Listening and Rating and continuing with Listening. The colored lines indicate the different Conditions: L (blue) = Listening, LR (red) = Listening and Rating. ^∗^Indicates a significant difference.

**FIGURE 3:**
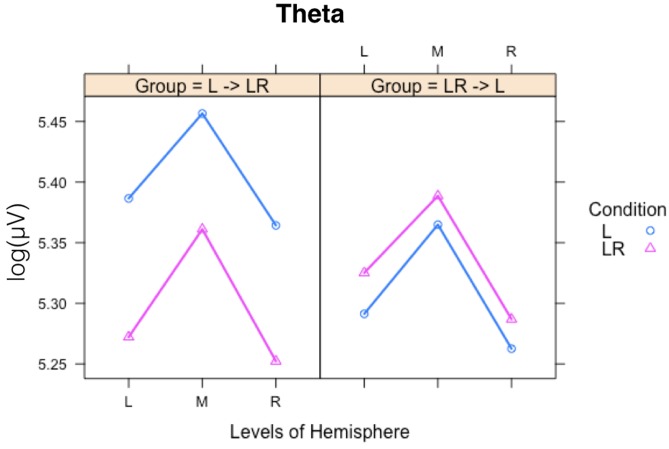
Interaction plot for the significant three-way Group × Condition × Hemisphere interaction for the theta band. Abscissa depicting the different Hemisphere positions (L = left, M = middle, R = right). The colored lines indicate the different Conditions: L (blue) = Listening, LR (red) = Listening and Rating. The left panel shows the results for the Group starting with Listening and Rating and continuing with Listening; the right panel shows the results for the Group starting with Listening and continuing with Listening and Rating.

**FIGURE 4:**
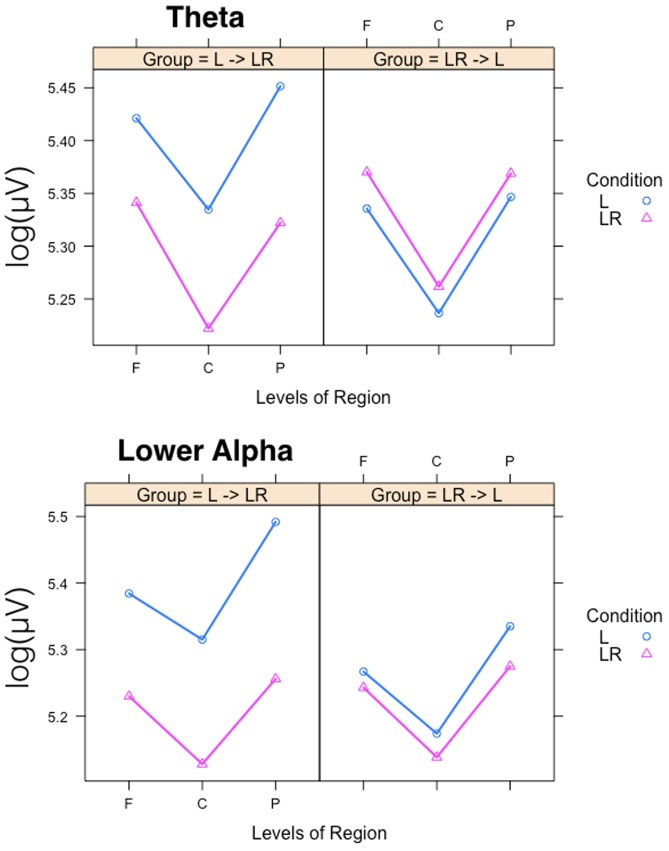
Interaction plots for the partly significant three-way Group × Condition × Region interactions for the theta and lower alpha band. Abscissa depicting the different positions in the fronto-parietal direction (F = frontal, C = central, P = parietal). The colored lines indicate the different Conditions: L (blue) = Listening, LR (red) = Listening and Rating. The left panel shows the results for the Group starting with Listening and Rating and continuing with Listening; the right panel shows the results for the Group starting with Listening and continuing with Listening and Rating.

Since, the **Group × Condition** interactions are most important for our study we will present them first. These interactions are graphically presented in **Figure 2**. *Post hoc* tests revealed stronger power values during the L condition than during the LR condition but only for those subjects who started with the L condition (Group L → LR). The other group who started with the LR condition showed basically similar power values during both conditions. There are also two partly significant three-way **Group × Condition × Region** interactions, which are graphically shown in **Figure 3**. These interactions are qualified by slightly stronger differences between L and LR for the central and parietal EOIs than for the frontal EOIs. There is also a three-way **Group × Condition × Hemisphere** interaction which became significant for the theta band power only **Figure 4**. This interaction is qualified by the stronger theta power values during the L than during the LR condition only for the subjects from the L → LR group. These L vs. LR differences are particularly strong for the central and parietal EOIs.

**FIGURE 5:**
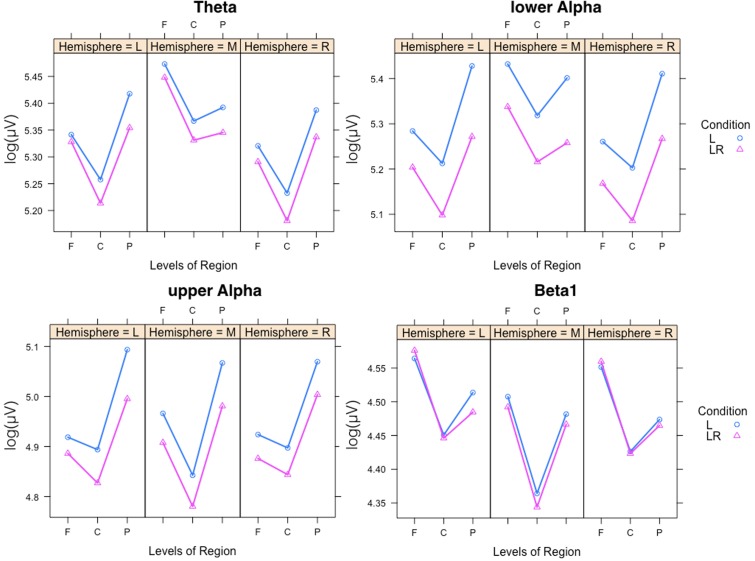
Significant three-way Condition × Hemisphere × Region interaction plots for the four frequency bands theta, lower alpha, upper alpha, and beta1. Abscissa depicting the different positions in the fronto-parietal direction (F = frontal, C = central, P = parietal). The colored lines indicate the different Conditions: L (blue) = Listening, LR (red) = Listening and Rating. The three panels of each figure depict the results for the left (L), middle (M), and right (R) part of the scalp.

In **Figure 5** the significant three-way **Condition × Hemisphere × Region** interaction plots are shown. As one can see from these plots the frontal to parietal profiles of the power values are different across the hemispheres and conditions. For lower and upper alpha, there are preponderances for the parietal EOI. For theta, the strongest power values are found for frontal midline EOIs. Beta1 power is mostly stronger frontally. In addition, the power values for the L condition are significantly stronger except for beta1. The two-way **Condition × Region** interactions revealed significant differences between the power values obtained during the L compared to the LR conditions for all regions and the four frequency bands except for the theta band at frontal EOIs.

**FIGURE 6:**
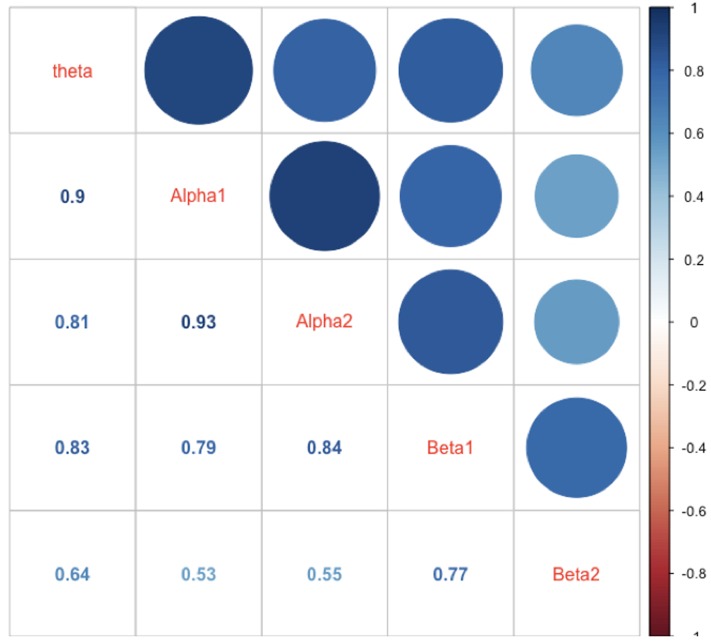
Correlations between mean power values of the different frequency bands. The correlations are represented as numbers, circles, and blue hue. The size of the circles as well as the saturation of the color indicates the size of the correlation (the larger the circle the larger the correlation, or the bluer the circle or the number the larger the correlations). The diagonal shows the name of the frequency bands.

In **Figure 6** the intercorrelations between the power values of the different frequency bands are shown. As one can see from this Figure the correlations are generally strong with the strongest correlation between adjacently located frequency bands. This speaks for a general mechanism driving and/or modulating the power in the different frequency bands.

## Discussion

In this study, we show that passive unconstrained music listening (L condition) is associated with increased power values in all examined frequency bands (theta, lower alpha, upper alpha, lower beta, upper beta). However, this power increase was only apparent when the subjects followed the L condition first. Thus, after following the LR condition first and the L condition second, the subjects obviously did not listen to the music during the L condition in an unconstrained manner. They most likely applied the same (or similar) mental state as used during the LR condition when listening to the music in the L condition. Possibly, they implicitly rated the musical pieces although they are not required to do so.

We also observed HR increases during the L condition but HR decreases during the LR condition. HR decreases are often reported during the performance of long-lasting and partly boring tasks, while HR increases are generally observed during emotionally arousing situations (i.e., during passive listening of emotionally arousing music) (Koelsch and Jancke, 2015). We interpreted the HR increase during the L condition as emotional arousal, which is evoked by listening to positively valenced music. The HR decrease during the LR condition might indicate less emotional arousal. For the EDA measures, the findings are less clear, as we obtained only a marginally significant effect for the Group × Condition interaction. However, this marginally significant interaction fits the general findings of our study. The subjects who began with the L condition demonstrated decreased EDA responses (indicating less vegetative arousal) during the LR condition. Thus, simultaneous music listening and rating appears less emotionally arousing after unconstrained music listening. In summary, the findings for both vegetative measures may indicate that simultaneous listening and rating is associated (at least slightly) with less emotional/vegetative arousal.

Nevertheless, what is the functional meaning of our findings? An initial approach to interpreting our findings is interpreting the power in the EEG frequency bands as indicators of the activity of different cortical and subcortical networks. The best-elaborated frequency band in this context is the alpha band. Meanwhile, several studies support the notion that alpha band oscillations are strongly related to cortical inhibition, especially of task-irrelevant brain areas (Klimesch et al., 2007). It has also been argued that alpha band oscillations indicate a neurophysiological process during which the engaged neural networks need to maintain optimal neural activation. This is most likely realized by maintaining an optimal level of excitation–inhibition through the suppression of neural networks, which might “disturb” or “interfere” with the on-going processing of the relevant task. This is consistent with findings demonstrating strong alpha power increases during such processes, which are characterized by redirecting attention from external events to internal thoughts. Typical examples of such psychological processes are meditation (Aftanas and Golocheikine, 2001; Faber et al., 2012); effortful cognition, such as perception of degraded speech (Weisz et al., 2011); imagination (Cooper et al., 2003; Schaefer et al., 2011b); creative thinking (Benedek et al., 2011); working memory tasks (Klimesch, 1999; Palva and Palva, 2007); or during unconstrained music listening (Iwaki et al., 1997; Schaefer et al., 2011b; Jancke et al., 2015). Previously, we speculated that this could indicate a “dragging into the music” while simultaneously neglecting other stimuli or even thoughts (Jancke et al., 2015). Thus, subjects let their minds wander per the musical rhythm, harmonies, and/or melody. In a way, this is similar to what occurs during meditation or imagination, psychological states that are also accompanied by increased alpha band power.

Explaining the functional meaning of the other frequency bands during music listening is less easy and straightforward. In our study, we also identified an increase in theta band power. Increased theta band power has been associated with three psychological functions: (a) the so-called frontal midline theta (Fm theta), which is generally related to cognitive effort, working memory, and emotion processing (Gevins et al., 1997; Sammler et al., 2007; Maurer et al., 2015; Wisniewski et al., 2015); (b) the widespread theta most prominent at the frontal and parietal scalp locations, which is associated with low-level alertness, drowsiness, and “mind-wandering” (Braboszcz and Delorme, 2011; Platt and Riedel, 2011; Baumeister et al., 2012; Park et al., 2014; Poudel et al., 2014); and (c) the widespread theta with parietal dominance, which has been related to the effective encoding of new memories (Klimesch, 1999). Theta increases have also been reported during meditation (Lagopoulos et al., 2009; Baijal and Srinivasan, 2010; Faber et al., 2012). The theta band power increase in our study was more widespread but also with a strong power increase over frontal midline EOIs. The musical pieces used in our study were all positively evaluated, thus it is possible that the frontal midline theta power increases observed were due to positively valenced arousal, as in the study of Sammler et al. (2007). It is also possible that the subjects exerted more mental effort to perceive and process the musical pieces. However, we find this explanation implausible, as the LR condition is definitively more demanding than the L condition. Thus, we would anticipate a greater workload and therefore more frontal midline theta during the LR condition than during the L condition. As we have argued previously, it is more plausible that our subjects were “drawn into” the music, resulting in a state that shares some similarities with the “mind-wandering” state, a state during which theta increases have frequently been reported (Braboszcz and Delorme, 2011). Mind-wandering is characterized by the experience of one’s attention drifting from a task or from external matters toward internal, mostly personal, issues. Braboszcz and Delorme (2011) argue that mind-wandering is a form of low-alertness and low-concentration state of rest.

Increased beta power during unconstrained music listening is, on one hand, difficult to explain, as tonic beta band power increases are generally reported during conditions of increased tonic alertness. During the resting state, upper alpha and beta band power both positively correlate with tonic alertness network activity (Sadaghiani et al., 2010). Thus, it is possible that the subjects were tonically alert (but with a more internal direction of attention) during the L condition. Increased beta band power has also been reported during emotional processing (Sebastiani et al., 2003; Aftanas et al., 2006), which is consistent with the fact that the musical pieces we used are also emotionally arousing. Increases in beta oscillations have also been observed during the “minimally conscious state” (MCS; Schiff et al., 2014). This state is characterized by intermittent or inconsistent evidence of consciousness. Typical behavioral signs are intermittent or inconsistent responses to verbal commands, reduced verbal output or object use, as well as intermittent or inconsistent purposeful eye movements. In terms of the EEG pattern, MCS is characterized by a coupled increase in theta and beta oscillations, which is thought to indicate functional or structural deafferentation of the thalamus from its cortical inputs.

One striking finding is that the power values in all examined frequency bands in our study were highly intercorrelated, suggesting strong functional interplay between the frequency bands (see, for example, Steriade, 2006). One reason could be that the different cognitive processes and associated networks, and their specific frequency bands, are simultaneously or sequentially active. Thus, when calculating power values for the frequency bands across longer periods (as we have done), it is most likely that they correlated substantially during the experiment. A further possibility is that all other frequencies are “grouped” around the alpha band oscillation according to harmonic rules (Klimesch, 2012). It has been suggested that this so-called “harmonic coupling” provides an optimal basis for a functional interchange between two or more oscillatory systems (Klimesch, 2012). In addition, we also should take into account the fact that the definition of the frequency bands used in the present study is somewhat arbitrary (although we used a traditional frequency band definition that has been used in most EEG studies so far), especially considering the borders between adjacent frequency bands. Thus, it is most likely that some overlap and leakage between the adjacently located band borders caused spurious correlations between the frequency bands.

However, we propose a further possibility for explaining the general power increase in all examined frequency bands during the L condition. It is possible that the active networks are highly synchronized and are not disrupted by interfering psychological processes. Thus, they can synchronize without any disturbance because they do the same during the entire music-listening process. Consequently, all networks oscillate synchronically, resulting in generally increased power values in several frequency bands. In future studies and analyses, it would thus be interesting to study the functional network architecture and the associated coherences during music listening. Some first studies have shown changed (mostly increased) coherences during unconstrained music listening (e.g., Bhattacharya and Petsche, 2001).

Our most important finding, however, is that the pattern of brain activation during unconstrained and undisturbed music listening depends on whether the subjects had followed the L condition before or after the LR condition. The LR condition is a multitasking condition wherein subjects were required to listen to the music, evaluate their emotional feelings, transfer these feelings to reportable (verbal) categories, and to indicate these feelings with the appropriate motor output. These psychological functions obviously disrupt unconstrained music listening and the associated brain activations. As mentioned above, we speculate that during unconstrained music listening, the subjects employed increased internal attention, accompanied by reduced external attention, increased inhibition of brain networks uninvolved in generating this internal state, and a mind-wandering state. Thus, subjects engaged in the LR condition could not let their mind wander; rather, they had to focus their attention on the manipulandum with which they indicated their internal state. They also focused their attention on their feelings and on the processes necessary for transferring the feelings to reportable categories. These different processes would prevent the development of mind-wandering as in unconstrained music listening. Interestingly, when the subjects were confronted with this particular state, it was obviously not that easy to switch to the unconstrained listening style.

A possible limitation of our study is that we did not explicitly examine whether repeated exposure to the L and LR conditions (i.e., L → L and LR → LR) might have revealed similar or different results to the L → LR and/or LR → L conditions. However, we refrained from using these conditions in our experiment because: (1) Previously (Jancke et al., 2015), we showed that during repeated presentation of a musical piece, with the same listening context, the EEG and vegetative responses are strikingly similar across the repeated presentations. Thus, we are certain that the neurophysiological and vegetative responses would have been the same across the repeated presentation of the L and LR conditions. (2) Including the abovementioned conditions would have increased the duration of the entire experiment for each subject. As our plan was to study the neurophysiological and vegetative responses to musical pieces in an ecologically valid setting, we wanted to keep the subjects’ experimental load as low as possible. Nevertheless, we are confident that we have shown that the neurophysiological and vegetative reactions to musical pieces depends, at least partly, on the listening context.

Taken together, we show that the power in all examined frequency bands increases during the L condition, but only when subjects have not had previous experience with the LR condition. During the LR condition, the power values are all substantially lower. We interpret the association between unconstrained music listening and increased power in all examined frequency bands as a neural indicator of a mental state, which can best be described as a mind-wandering state, during which subjects are “drawn into the music.” Further studies are needed to delineate possible characteristic differences (if they exist) between what has typically been described as mind-wandering or meditation.

## Author Contributions

LJ designed the experimental paradigm, performed the statistical analysis and drafted the manuscript. AM conducted the experiments, did the analysis of the EEG, vegetative, and behavioral data, and drafted the manuscript. JK designed the experimental paradigm, performed the statistical analysis and drafted the manuscript. All authors read and approved the final manuscript.

## Conflict of Interest Statement

The authors declare that the research was conducted in the absence of any commercial or financial relationships that could be construed as a potential conflict of interest.
