# Supplementary material for: Task Context Influences Brain Activation during Music Listening
Source: Front Hum Neurosci. 2017 Jun 29;11:342. doi: 10.3389/fnhum.2017.00342 (PMC5489556; doi:10.3389/fnhum.2017.00342)
Supplement: Supplementary file 1 [file Table_1.DOCX]

# Appendix – ANOVA Tables

**Theta**

|Effect |df |MSE |F |ges |p.value |

|:---------------------------------|:------------|:--------|:---------|:------|:-------|

|Group |1, 49 |50174.52 |0.22 |.003 |.64 |

|Condition |1, 49 |5679.82 |4.08 * |.007 |.05 |

|Group:Condition |1, 49 |5679.82 |9.59 ** |.02 |.003 |

|Hemisphere |1.98, 97.20 |2297.84 |19.20 *** |.03 |<.0001 |

|Group:Hemisphere |1.98, 97.20 |2297.84 |0.00 |<.0001 |>.99 |

|Region |1.82, 89.23 |1507.23 |32.55 *** |.03 |<.0001 |

|Group:Region |1.82, 89.23 |1507.23 |0.02 |<.0001 |.97 |

|Condition:Hemisphere |1.95, 95.53 |94.30 |0.74 |<.0001 |.47 |

|Group:Condition:Hemisphere |1.95, 95.53 |94.30 |1.84 |.0001 |.16 |

|Condition:Region |1.82, 88.98 |155.76 |7.81 ** |.0007 |.001 |

|Group:Condition:Region |1.82, 88.98 |155.76 |5.48 ** |.0005 |.007 |

|Hemisphere:Region |3.04, 148.90 |1050.87 |14.55 *** |.01 |<.0001 |

|Group:Hemisphere:Region |3.04, 148.90 |1050.87 |0.53 |.0005 |.67 |

|Condition:Hemisphere:Region |3.28, 160.48 |51.37 |2.49 + |.0001 |.06 |

|Group:Condition:Hemisphere:Region |3.28, 160.48 |51.37 |0.56 |<.0001 |.66 |

**Alpha**-**1**

|Effect |df |MSE |F |ges |p.value |

|:---------------------------------|:------------|:--------|:---------|:------|:-------|

|Group |1, 49 |89060.08 |0.98 |.01 |.33 |

|Condition |1, 49 |20271.05 |11.73 ** |.04 |.001 |

|Group:Condition |1, 49 |20271.05 |4.30 * |.01 |.04 |

|Hemisphere |1.96, 96.13 |2170.99 |15.95 *** |.01 |<.0001 |

|Group:Hemisphere |1.96, 96.13 |2170.99 |0.17 |.0001 |.84 |

|Region |1.71, 83.81 |2306.68 |38.25 *** |.02 |<.0001 |

|Group:Region |1.71, 83.81 |2306.68 |0.64 |.0004 |.51 |

|Condition:Hemisphere |1.69, 82.90 |198.35 |1.14 |<.0001 |.32 |

|Group:Condition:Hemisphere |1.69, 82.90 |198.35 |1.04 |<.0001 |.35 |

|Condition:Region |1.18, 57.87 |806.78 |17.57 *** |.003 |<.0001 |

|Group:Condition:Region |1.18, 57.87 |806.78 |4.16 * |.0007 |.04 |

|Hemisphere:Region |2.96, 144.91 |961.54 |17.44 *** |.008 |<.0001 |

|Group:Hemisphere:Region |2.96, 144.91 |961.54 |1.27 |.0006 |.29 |

|Condition:Hemisphere:Region |2.57, 126.00 |117.14 |2.25 + |.0001 |.10 |

|Group:Condition:Hemisphere:Region |2.57, 126.00 |117.14 |0.56 |<.0001 |.62 |

**Alpha**-**2**

|Effect |df |MSE |F |ges |p.value |

|:---------------------------------|:------------|:---------|:---------|:------|:-------|

|Group |1, 49 |127903.42 |0.22 |.003 |.64 |

|Condition |1, 49 |25820.31 |15.99 *** |.05 |.0002 |

|Group:Condition |1, 49 |25820.31 |2.99 + |.009 |.09 |

|Hemisphere |1.93, 94.39 |1989.04 |2.83 + |.001 |.07 |

|Group:Hemisphere |1.93, 94.39 |1989.04 |0.42 |.0002 |.65 |

|Region |1.28, 62.69 |5428.64 |70.44 *** |.06 |<.0001 |

|Group:Region |1.28, 62.69 |5428.64 |0.33 |.0003 |.63 |

|Condition:Hemisphere |1.41, 68.87 |315.59 |6.02 ** |.0003 |.009 |

|Group:Condition:Hemisphere |1.41, 68.87 |315.59 |0.73 |<.0001 |.44 |

|Condition:Region |1.16, 56.71 |1803.45 |28.14 *** |.007 |<.0001 |

|Group:Condition:Region |1.16, 56.71 |1803.45 |1.63 |.0004 |.21 |

|Hemisphere:Region |2.65, 129.84 |1004.85 |8.66 *** |.003 |<.0001 |

|Group:Hemisphere:Region |2.65, 129.84 |1004.85 |2.05 |.0007 |.12 |

|Condition:Hemisphere:Region |2.27, 111.11 |227.12 |3.24 * |.0002 |.04 |

|Group:Condition:Hemisphere:Region |2.27, 111.11 |227.12 |0.20 |<.0001 |.85 |

**Beta**-**1**

|Effect |df |MSE |F |ges |p.value |

|:---------------------------------|:------------|:--------|:---------|:------|:-------|

|Group |1, 49 |29061.35 |0.00 |<.0001 |.99 |

|Condition |1, 49 |1605.48 |15.05 *** |.01 |.0003 |

|Group:Condition |1, 49 |1605.48 |16.70 *** |.02 |.0002 |

|Hemisphere |1.90, 92.91 |978.81 |0.21 |.0002 |.80 |

|Group:Hemisphere |1.90, 92.91 |978.81 |0.05 |<.0001 |.95 |

|Region |1.35, 66.13 |1064.51 |82.60 *** |.06 |<.0001 |

|Group:Region |1.35, 66.13 |1064.51 |0.09 |<.0001 |.83 |

|Condition:Hemisphere |1.66, 81.16 |47.92 |4.00 * |.0002 |.03 |

|Group:Condition:Hemisphere |1.66, 81.16 |47.92 |1.74 |<.0001 |.19 |

|Condition:Region |1.71, 83.61 |78.75 |17.67 *** |.001 |<.0001 |

|Group:Condition:Region |1.71, 83.61 |78.75 |1.60 |.0001 |.21 |

|Hemisphere:Region |3.38, 165.61 |351.09 |4.55 ** |.003 |.003 |

|Group:Hemisphere:Region |3.38, 165.61 |351.09 |0.82 |.0006 |.50 |

|Condition:Hemisphere:Region |2.87, 140.84 |27.12 |5.49 ** |.0002 |.002 |

|Group:Condition:Hemisphere:Region |2.87, 140.84 |27.12 |0.97 |<.0001 |.41 |

**Beta**-**2**

|Effect |df |MSE |F |ges |p.value |

|:---------------------------------|:------------|:-------|:---------|:------|:-------|

|Group |1, 49 |6085.20 |0.81 |.01 |.37 |

|Condition |1, 49 |578.19 |0.31 |.0004 |.58 |

|Group:Condition |1, 49 |578.19 |22.26 *** |.03 |<.0001 |

|Hemisphere |1.88, 91.96 |610.96 |4.97 * |.01 |.01 |

|Group:Hemisphere |1.88, 91.96 |610.96 |0.11 |.0003 |.88 |

|Region |1.78, 87.10 |425.01 |29.60 *** |.04 |<.0001 |

|Group:Region |1.78, 87.10 |425.01 |1.32 |.002 |.27 |

|Condition:Hemisphere |1.99, 97.41 |41.65 |1.18 |.0002 |.31 |

|Group:Condition:Hemisphere |1.99, 97.41 |41.65 |2.42 + |.0004 |.09 |

|Condition:Region |1.51, 74.16 |63.20 |2.17 |.0004 |.13 |

|Group:Condition:Region |1.51, 74.16 |63.20 |0.31 |<.0001 |.68 |

|Hemisphere:Region |3.09, 151.49 |306.78 |1.67 |.003 |.17 |

|Group:Hemisphere:Region |3.09, 151.49 |306.78 |0.22 |.0004 |.88 |

|Condition:Hemisphere:Region |3.23, 158.15 |22.18 |1.81 |.0003 |.14 |

|Group:Condition:Hemisphere:Region |3.23, 158.15 |22.18 |1.18 |.0002 |.32 |
